# An update on the roles of immune system-derived microRNAs in cardiovascular diseases

**DOI:** 10.1093/cvr/cvab007

**Published:** 2021-01-23

**Authors:** Luke B Roberts, Puja Kapoor, Jane K Howard, Ajay M Shah, Graham M Lord

**Affiliations:** 1 School of Immunology and Microbial Sciences, King’s College London, Great Maze Pond, London SE1 9RT, UK; 2 School of Cardiovascular Medicine and Sciences, King’s British Heart Foundation Centre, King’s College London, 125 Coldharbour Lane, London SE5 9NU, UK; 3 School of Life Course Sciences, King’s College London, Great Maze Pond, London SE1 9RT, UK; 4 Faculty of Biology, Medicine and Health, University of Manchester, 46 Grafton Street, Manchester M13 9NT, UK

**Keywords:** Heart, Cardiovascular disease, Cardioimmunology, Immunology, MicroRNA, TREGS

## Abstract

Cardiovascular diseases (CVD) are a leading cause of human death worldwide. Over the past two decades, the emerging field of cardioimmunology has demonstrated how cells of the immune system play vital roles in the pathogenesis of CVD. MicroRNAs (miRNAs) are critical regulators of cellular identity and function. Cell-intrinsic, as well as cell-extrinsic, roles of immune and inflammatory cell-derived miRNAs have been, and continue to be, extensively studied. Several ‘immuno-miRNAs’ appear to be specifically expressed or demonstrate greatly enriched expression within leucocytes. Identification of miRNAs as critical regulators of immune system signalling pathways has posed the question of whether and how targeting these molecules therapeutically, may afford opportunities for disease treatment and/or management. As the field of cardioimmunology rapidly continues to advance, this review discusses findings from recent human and murine studies which contribute to our understanding of how leucocytes of innate and adaptive immunity are regulated—and may also regulate other cell types, via the actions of the miRNAs they express, in the context of CVD. Finally, we focus on available information regarding miRNA regulation of regulatory T cells and argue that targeted manipulation of miRNA regulated pathways in these cells may hold therapeutic promise for the treatment of CVD and associated risk factors.

## 1. Introduction

Cardiovascular disease (CVD) is the leading cause of pre-mature death with ∼17.8 million deaths worldwide in 2017.^1^ With approximately half a billion people afflicted by CVD globally, it also constitutes a major socioeconomic burden, accruing substantial direct costs to healthcare systems as well as indirect costs of reduced productivity and days of work lost to illness.^1^

The heart and vasculature are primarily composed of non-haematopoietically derived cell types including cardiomyocytes, fibroblasts, endothelial cells (ECs), smooth muscle cells, pericytes, and stroma. However, the presence of leucocytes within cardiovascular tissue has long been acknowledged and all the major immune cell subtypes, including lymphocyte and myeloid-derived populations, are found within cardiovascular tissues.^2^^,^^3^ Some of these populations are resident cells, while infiltrating immune cells contribute to changes in the structural and functional properties of the heart and vasculature and may amplify CVD progression. Over the last two decades, the broad roles played by cells of the immune response in cardiovascular biology have begun to be appreciated. These range from roles in heart development, to maintenance of vascular homoeostasis and steady state physiology through to critical roles in directing and protecting from disease-related pathology. Interested readers are directed to specialized recent reviews on the field, provided by Swirski and Nahrendorf^2^ and Strassheim *et al*.^4^

Understanding the critical regulators of immune cell activity and function is key to appreciate how immune responses which underlie or exacerbate certain diseases might be corrected to cure disease. MicroRNAs (miRNAs) are among the most important regulators of immune cellular function and identity. This review aims to provide an up-to-date discussion of available literature, which investigates immune cell-derived miRNAs and their roles in regulating immune responses in the setting of CVD and its associated risk factors. We additionally highlight putative roles played by immune system-derived miRNAs delivered to non-immune cardiovascular cell types via extracellular vesicles (EVs). Finally, we discuss miRNA regulation of regulatory T cells (T_REGS_) and suggest that the manipulation of miRNA pathways in these cells may prove beneficial in the context of T_REGS_ immunotherapy and CVD. A reference for abbreviations used throughout this manuscript can be found in Table 1.

**Table 1:** Meanings of abbreviations used in this review

Abbreviation	Meaning
AT	Adipose tissue
CAD	Coronary artery disease
CVD	Cardiovascular disease
DCM	Dilated cardiomyopathy
EAM	Experimental autoimmune myocarditis
EAT	Epicardial adipose tissue
ECs	Endothelial cells
EVs	Extracellular vesicles
FC	Foam cells
HF	Heart failure
LAEAT	Left atrial epicardial adipose tissue
MI	Myocardial infarction
Th	CD4^+^ helper T cell
T_REGS_	CD4^+^ regulatory T cells

## 2. Immune system enriched miRNAs: roles in immune cell development and function

**Figure 1:**
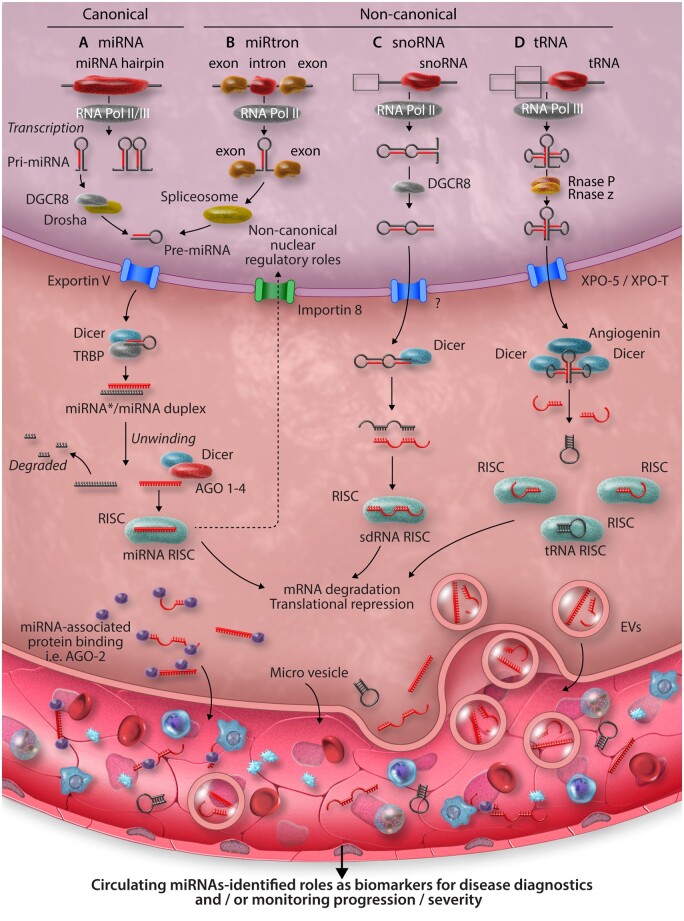
Overview of mammalian miRNA biogenesis pathways and functions. (*A*) Canonically, microRNAs are generated following RNA polymerase II/III dependant transcription as primary transcripts (pri-miRNAs). Cleavage of the pri-miRNA to a stem-loop, pre-miRNA follows within the nucleus, orchestrated by the microprocessor complex comprising the RNAse-III like enzyme, Drosha, and a dsDNA binding co-factor—DiGeorge syndrome chromosomal region 8 (DGCR8). Next, pre-miRNAs are translocated to the cytoplasm via the RAS-related nuclear protein-guanosine-5′-triphosphate-ase (Ran-GTP) dependant, Exportin V nuclear transporter. A final cleavage of pre-miRNA to a duplex containing the mature microRNA sequences is undertaken by Dicer. Dicer also integrates the duplex within an Argonaut protein family member (AGO1-4 in mammals) wherein, one strand of the duplex is typically degraded, while the other is selected to remain AGO-associated, forming the microRNA-induced silencing complex (miRISC). It is the miRISC, which facilitates localization and binding to mRNA target sequences. Finally, following formation of the miRISC: mRNA complex, enzymatic degradation of the mRNA transcript or its direct translational inhibition occurs, epigenetically altering gene expression by modifying the dynamics of protein synthesis. While all mature microRNAs are thought to be generated within the cytoplasm, nuclear import of AGO2 loaded with microRNA can occur in an Importin 8-dependant manner and described nuclear functions of microRNAs includes post-transcriptional gene-silencing, transcriptional gene-silencing, and transcriptional gene activation, via binding to recently transcribed RNAs, or through direct chromatin interactions with gene promotor or enhancer regions.^6–8^ (*B*) MiRtrons are derived from sequences spliced within the introns of coding genes. In the nucleus, processing of miRtrons bypasses the requirement for conventional Drosha/DGCR8 cleavage. The pre-miRNA is derived from spliceosome activity.^9^^,^^10^ (*C*) Small nucleolar RNAs undergo splicing and debranching in the nucleus before being exported to the cytoplasm via an unknown nuclear transporter. Here, they undergo final cleavage by Dicer before being incorporated into the RISC. (*D*) RNA polymerase III transcribed tRNA undergo processing by RNases, which cleave the 5′ and 3′ ends allowing its transport into the cytoplasm via Exportin-t. tRNAs undergo further cleavage by Dicer and Angiogenin before becoming incorporated into the tRNA RISC.^11^ Mature miRNAs can be released into the circulation via the excretion of EVs, exosomes, budding of microvesicles, or via proteins. In the circulation they can be readily detected in biological fluids, such as blood, urine, and saliva, thus acting as biomarkers of disease, creating unique miRNA signatures in response to disease.

MicroRNAs play essential roles in governing immune system development, homoeostasis, and activation.^5^ The canonical pathways of miRNA biogenesis are well recognized but more recently, non-canonical biogenesis pathways and novel miRNA regulatory mechanisms have also been described (*Figure 1*). Dysregulated expression of miRNAs during disease settings including CVD, autoimmunity, and tumourigenesis has been extensively reported,^12^^,^^13^ and alterations to the expressions of many of these miRNAs may serve useful roles as biomarkers for diagnostic purposes and/or for monitoring disease progression.^13^ Many individual miRNAs highly expressed by immune system cells—the so-called ‘immuno-miRNAs’, play unique roles in directing immune system responses and additional novel players are often described. However, some of these serve particularly critical roles and warrant a brief introduction.

### 2.1 miRNA-17-92 cluster

The cluster encodes six unique miRNAs: miR-17, miR-18a, miR-19a, miR-20a, miR-19b-1, and miR-92a-1 and plays multiple, non-redundant roles in B cell development, supportive of its actions as an oncogene promoting B cell lymphomas.^14^ Other critical roles include regulation of T cell activation, maturation and development of T-helper subsets (Th) and T_REGS_, and additional important functions in monocyte differentiation to macrophages and macrophage activation pathways.^15^

### 2.2 miRNA-21

Expressed in multiple cell types in addition to leukocytes, up-regulation of miR-21 (referring to the predominantly expressed mature strand miR-21-5p) is associated with exposure to inflammatory stimuli and immune cell activation.^16^ Additionally, miR-21 functions as an oncogene and its overexpression results in B cell lymphoma in mice,^17^ supporting the association of miR-21 with the development of many cancers.^18^ Aging is associated with increased expression of miR-21 in T cells, resulting in failure to generate memory T cells, contributing to aging-related immune system dysfunction (immunosenescence).^19^

### 2.3 miRNA-142

miRNA-142 is a highly evolutionarily conserved miRNA, widely considered to be haematopoietic-lineage specific or enriched.^20^^,^^21^ Expression of mature isoforms of this miRNA from both arms of the miR-142 precursor (miR-142-3p and miR-142-5p) has been extensively validated. Multiple roles in the development of haematopoietic lineages and immune system function are attributed to these molecules.^22–28^ The regulation of lineage-enriched expression of miR-142 isoforms may be related to the association of *MIR142* promotor activity with super-enhancers which are predominantly active in haematopoietic and immune cell types^29^ and epigenetic factors, such as lineage and/or context-specific differences in DNA methylation state of the *MIR142* promotor.^30^^,^^31^

### 2.4 miRNA-146a

miRNA-146a is a powerful negative regulator of both innate and adaptive immune responses.^32–34^ Expression of miR-146a is driven by the NF-kB signalling pathway^35^ and is correlated with immune cell activation via T cell receptor signalling or Toll-like receptor activation. Critically, miR-146a-5p functions as a negative feedback regulator, down-regulating NF-kB signalling transducers IRAK1 and TRAF6 to limit cellular activation and its own expression levels.^33^^,^^34^

### 2.5 miRNA-155

miRNA-155 is a master regulator of immune system responses. Mice deficient for miR-155 have severely defective lymphoid and myeloid cell development and homoeostasis.^36^^,^^37^ Activation of immune cells is additionally promoted by miR-155.^38^ Often, this has been linked to repression of pathways involving the canonical miR-155-5p target, suppressor of cytokine signalling 1 (SOCS1), a potent inhibitor of Janus kinase (JAK)-signal transducer and activator of transcription (STAT) signalling pathways.^39–41^

### 2.6 miRNA-181

Demonstration that miR-181 is highly and specifically expressed in haematopoietic progenitor cells gave one of the first indications that miRNAs might play critical roles in immune cell lineage differentiation.^20^ Subsequently, miR-181 family members (miR-181a-1, miR-181a-2, miR-181b-1, miR-181b-2, miR-181c, and miR-181d) have been shown to play critical roles in lymphoid and myeloid lineage differentiation and act as a metabolic rheostat for the immune system.^42–44^ Down-regulation of miR-181 family member expression may underlie reduced or dysregulated immune system function associated with aging and immunosenescence.^45^^,^^46^

### 2.7 miRNA-223

Particularly critical in the regulation of innate immunity,^47^^,^^48^ expression of miR-223 within the immune system is restricted largely to myeloid-derived cell types, in which miR-223-3p acts as a critical mediator of both myeloid cell differentiation and activation.^47^

## 3 miRNA-mediated regulation of immunity and CVD risk factors

### 3.1 Hypertension

While immune cell contribution to hypertension was discovered many years ago, it was not until the mid-2000s that a ground-breaking study by Guzik *et al*. showed that *Rag1*^−/−^ mice lacking T and B cells were protected against angiotensin II (Ang-II) and deoxycorticosterone acetate salt-induced hypertension. Moreover, the adoptive transfer of T but not B cells was able to restore the hypertensive abnormalities seen in the control groups, suggesting that T cells are critical mediators of hypertension.^49^

In a murine model of Ang-II-induced hypertension, levels of the miR-199/214 family member miR-214-3p were up-regulated eight-fold in perivascular tissue of Ang-II-infused mice compared to wild type (WT) mice. Moreover, under Ang-II infusion, miR-214^−/−^ mice showed blunted hypertension, vascular stiffening, and perivascular fibrosis when compared with WT controls. These effects mapped specifically to changes in the T cell transcriptome in the miR-214 ^−/−^ mice, which led to blunted T cell activation and infiltration into perivascular adipose tissue (AT). To assess the translational relevance of this, it was found that plasma miR-214-3p expression levels were increased in hypertensive patients.^50^ In another murine study of Ang-II-induced hypertension, the knockout of miR-31 (miR-31^−/−^), which maps specifically to T_reg_ and Th_17_ cells led to blunted hypertension and decreased cardiac hypertrophy and fibrosis. Furthermore, miR-31 ablation in mice resulted in a decrease of kidney infiltrating CD45^+^F4/80^+^ macrophages and an increase in kidney specific CD4^+^FoxP3^+^T_REGS_. As a result, miR-31^−/−^ mice had reduced vascular and renal damage. These differences mapped specifically to miR-31^−/−^ mediated post-transcriptional regulation of its direct target protein phosphatase 6c.^51^

### 3.2 Obesity and epicardial AT

Obesity and diabetes are major risk factors for CVD. AT is an endocrine organ of paramount importance in the development of systemic inflammation, which affects cardiovascular cell metabolism, function, and disease progression. Furthermore, the immune system plays critical roles in the development and function of AT and obesity is increasingly considered to be an immunometabolic disease. Thickening of epicardial adipose tissue (EAT), which surrounds the heart and major vessels is associated with coronary artery disease (CAD). In patients with sudden-CAD associated death, levels of miR-34a-3p and miR-34a-5p in EAT were up-regulated.^52^ This is of particular interest as miR-34a has been associated with obesity-induced systemic inflammation via the suppression of anti-inflammatory ‘M2’ macrophage polarization and increase in pro-inflammatory ‘M1’ macrophages and cytokines within visceral AT of murine models of diet-induced obesity.^53^ Furthermore, whole-genome sequencing on EAT from CAD patients, shows that genes involved in antigen presentation, chemokine signalling, and systemic inflammation are up-regulated, whilst levels of miR-103-3p, which regulates Th_2_ chemokines, like CCL13, were down-regulated.^54^ Interestingly, the presence of EAT around the left atria (LAEAT) has been linked to the prevalence of atrial fibrillation. Using data from the miRhythm study and bioinformatic platforms, Tran *et al.* analysed miRNA levels associated with LAEAT from AF patients. Levels of miR-155-5p and miR-302a-3p were associated with LAEAT in AF. Both miRNAs are involved in the regulation of IL-8, which has been linked to leukocyte trafficking and activation.^55^^,^^56^ Furthermore, downstream predicted genes of both miRNAs were associated with cardiac hypertrophy and adipogenesis.^4^

## 4. miRNA-mediated regulation of immune cells during CVD and associated complications

### 4.1 Atherosclerosis

**Figure 2:**
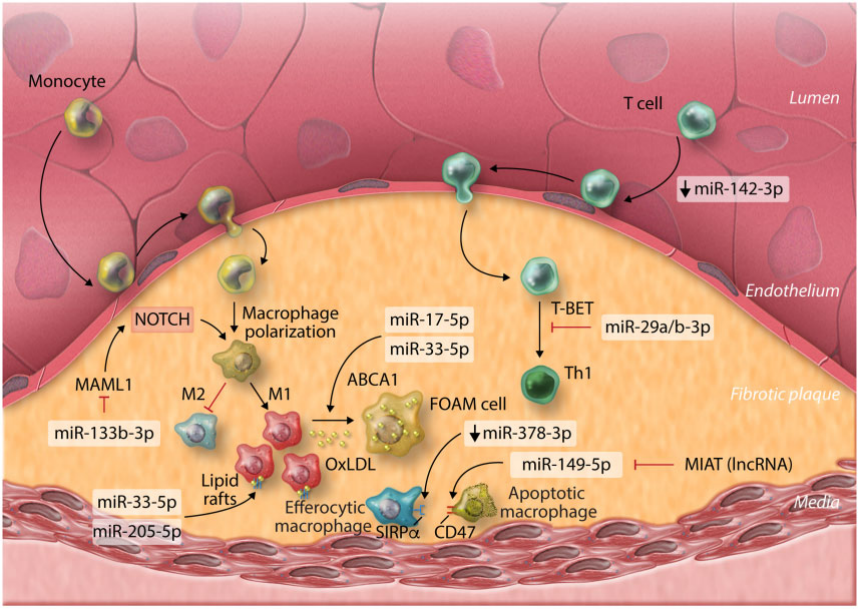
MicroRNAs involved with plaque-associated immune cells in atherosclerosis and their functions. OxLDL, oxidized low-density lipoprotein; lncRNA, long non-coding ribonucleic acid; MIAT, myocardial infarction associated transcript.

Atherosclerosis is considered to be an inflammatory, immune-mediated disease. Of the immune cells associated with atherosclerosis pathogenesis, macrophages play a central role in plaque formation and disease progression. As LDL retained beneath the endothelial wall becomes oxidized (oxLDL), it activates ECs and promotes the recruitment and infiltration of monocytes/macrophages. Uptake of oxLDL and internalization of apoptotic cell fragments by macrophages results in lipid accumulation and foam cell (FC) formation. The roles of miRNAs in the regulation of macrophages during atherosclerosis have been extensively investigated.^57^ Recent work has continued to uncover novel miRNA-mediated mechanisms of macrophage regulation and their athero-protective and atherogenic effects (*Figure 2*). Utilizing publicly available miRNA array data, Zheng *et al.*^58^ postulated that miR-133b-3p expression in macrophages serves a pro-atherogenic role by modulating expression of mastermind-like protein 1 (MAML1), tentatively supporting studies which highlight a role for NOTCH signalling in plaque leukocyte influx and atherosclerosis progression.^59–61^ However, although the authors convincingly demonstrated regulation of MAML1 by miR-133b-3p, given the well-appreciated role of MAML1 as a transcriptional co-activator of NOTCH signalling, it is not immediately clear how de-repression of MAML1 expression via down-regulation of miR-133b-3p might promote the associated down-regulation of various NOTCH pathway components—as implied by the authors. Therefore, further work which clarifies the exact relevance of the miR-133b–3p/MAML1 interaction in macrophages during atherosclerosis is required.

miRNA-17-5p—a potentially useful, circulating biomarker for monitoring atherosclerosis severity,^62^ has also been implicated in the regulation of inflammation and macrophage lipid accumulation during atherosclerosis, through the direct regulation of ATP-binding cassette transporter A1 (ABCA1), a critical component of cellular reverse cholesterol transport/lipid efflux.^63^ Down-regulation of ABCA1 leads to accumulation of intracellular lipids, promoting FC generation during atherosclerosis. miRNA-17-5p joins several additional miRNAs demonstrated to regulate ABCA1 in macrophages, thereby limiting reverse cholesterol transport,^57^^,^^64^ including the miR-33 family.^65^^,^^66^ miRNA-33-5p also regulates macrophage expression of peroxisome proliferator-activated receptor gamma co-activator 1-alpha, a master regulator of mitochondrial biogenesis and energy metabolism—therefore limiting cholesterol efflux via ABCA1 by restricting production of ATP.^67^ Inhibition of cholesterol efflux can promote formation of lipid-rich cell membrane rafts, promoting pro-inflammatory extracellular signalling pathways and inflammation.^68^ Alongside enhanced plaque instability, miR-205-5p was recently shown to direct lipid raft formation in macrophages, promoting pro-inflammatory macrophage phenotypes,^69^ expanding the understanding of miRNA-mediated control of macrophage lipid transport and its importance during atherosclerosis.

Defective macrophage clearance of other apoptotic macrophages and FCs (efferocytosis) may also underlie inflammation and atherosclerosis progression. Down-regulation of miR-378a-3p in aortic macrophages of hyperlipidemic *Apoe*^−/−^ mice was shown to result in de-repression of signal regulatory protein α (SIRPα),^70^ the transmembrane signalling receptor for CD47 (integrin-associated protein). The CD47: SIRPα axis act as a *don’t eat me* signal and up-regulation of this pathway prevents clearance of apoptotic cells within plaque lesions, accelerating atherosclerosis progression. Dysregulation of the CD47: SIRPα axis could also underlie atherosclerosis progression in humans, as was implied by the observation that the long non-coding RNA (lncRNA) myocardial infarction associated transcript (MIAT) was significantly higher in the circulating sera of atherosclerosis patients and those with symptoms of vulnerable atherosclerotic plaque, compared to controls, indicating that MIAT may also serve as a useful biomarker of plaque stability and atherosclerosis severity.^71^ In this study, the authors suggest that MIAT acts as a competing endogenous RNA (ceRNA) for miR-149-5p in macrophages and contains complementary miR-149-5p binding sequences which allow MIAT to ‘sponge’ miR-149-5p away from *CD47*, de-repressing CD47 expression. This study appears to convincingly demonstrate a functional role for this interaction, using ceRNA overexpression systems and *in vitro* miRNA manipulation to probe the nature of this dynamic in macrophage cell lines. However, the degree to which ceRNAs truly act to regulate endogenous miRNA activity through modulation of bioavailability for molecular target interaction is controversial (reviewed by Thomson and Dinger^72^). In their article, the authors do not investigate the limitations that physiological expression levels of both molecules may impose upon the outcome of the interaction during the real-world context of disease. This general criticism applies to many attempts to characterize cell-endogenous physiological regulators of miRNA activity. However, the concept presented in this work highlights the importance of considering the complex components of networks that regulate the functional activity of specific miRNAs in a given cell type.

Additional leukocytes also serve critical roles in the pathophysiology of atherosclerosis. T cells and particularly the CD4^+^ lineage are important players in atherosclerosis pathogenesis and the influx of CD4^+^ T cells to the vascular walls may be regulated by alterations to cytoskeleton dynamics as a result of aberrant expression of miR-142-3p.^73^ CD4^+^ Th_1_ responses, directed by the Th_1_ master T-box transcriptional regulators T-BET and (to some extent) Eomesodermin (EOMES) are the most prevalent Th subset in atherosclerotic plaques and promote atherosclerosis progression and severity.^74–76^ miRNA-29 seed-family members miR-29a-3p and miR-29b-3p act to restrict T-BET and EOMES expression in T cells^77^ and may therefore putatively alleviate atherosclerosis and enhance plaque stability by directly inhibiting Th_1_ development. However, evidence that global antagonism of miR-29 members using locked nucleic acids (LNAs) decreases atherosclerotic lesion size and promotes plaque remodelling and stability in mice,^78^ suggests that in certain cell types, miR-29 family members may actually contribute to disease progression. However, in this study, the impact of LNA-miR-29 on the development of Th1 responses was not specifically addressed. More studies are required to fully define the contribution of T-cell-derived miR-29 family members to the promotion of plaque-associated Th_1_ development, both during human and murine disease, in order to guide potential therapeutic strategies.

### 4.2 Myocardial infarction

**Figure 3:**
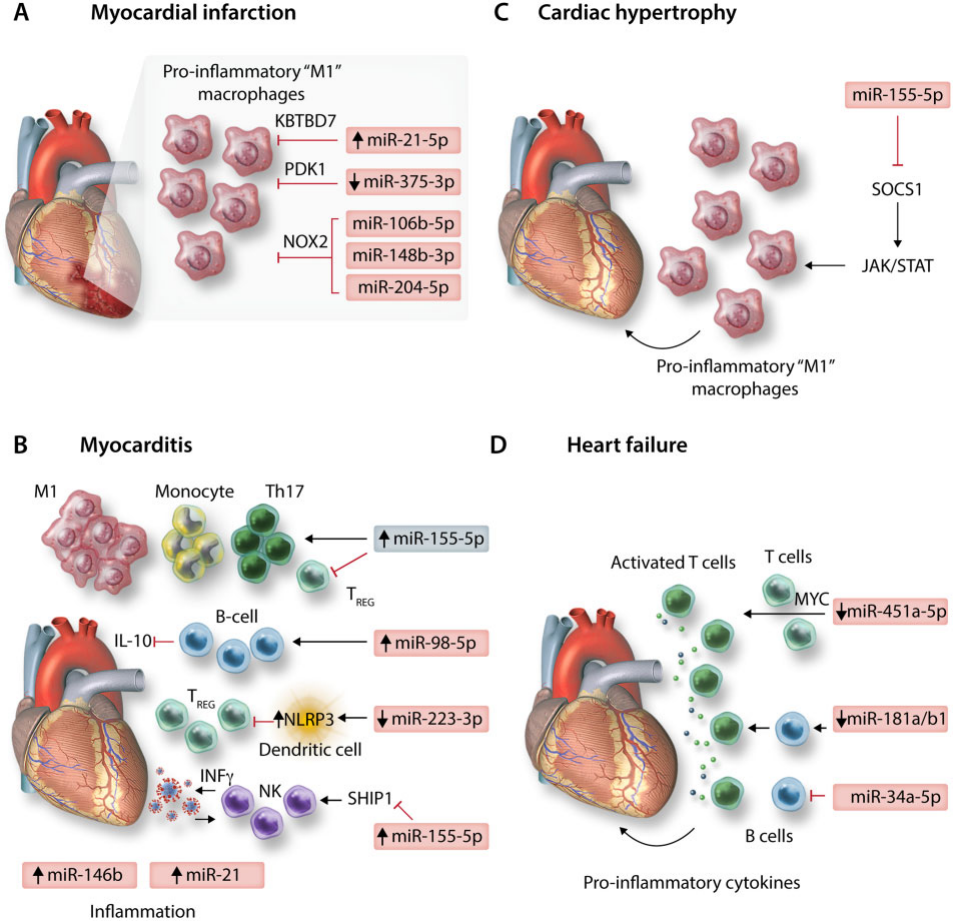
MicroRNAs involved in immune-regulation of diseases of the heart: depicting (*A*) myocardial infarction, (*B*) myocarditis, (*C*) cardiac hypertrophy, and (*D*) heart failure.

Monocytes and macrophages are critical mediators of inflammation and promote cardiomyocyte apoptosis during the early inflammatory response post-MI, but also contribute to later stages of left ventricular remodelling and dilation, eventually leading to HF.^79^ miRNA-21-5p may be a critical endogenous mediator, which limits the polarization of pro-inflammatory macrophages in the heart, post-MI. miRNA-21-5p expression was up-regulated rapidly in infarct areas of murine cardiac tissue, however, MI-induction in mice deficient for miR-21 (miR-21^−/−^) resulted in worse survival rates and greater infarct size and scarring.^80^ Macrophage expression of miR-21-5p directly restricted inflammatory activation and cytokine production by targeting Kelch repeat and BTB (POZ) domain containing 7 (KBTBD7), a transcriptional activator involved in mitogen-activated protein kinase signalling. In the absence of miR-21-5p, KBTBD7 overexpression enhanced macrophage activation by promoting p38 and NF-kB activation.^80^ Promotion of miR-21 regulatory mechanisms through intravenous delivery of nanoparticles (NPs) containing miR-21-5p mimics, targeted to cardiac macrophages shortly after MI-induction may therefore be a therapeutic strategy to limit pro-inflammatory/promote reparative, wound healing macrophage phenotypes, leading to attenuation of pathological remodelling.^81^ Therapeutically targeting miR-375-3p in macrophages may be an additional means of modulating pro-inflammatory macrophage development post-MI, via activation of 3-phosphoinositide-dependent protein kinase 1 and reduction of pro-inflammatory cytokine expression.^82^ Additionally, expression of a superoxide producing enzyme, NADPH oxidase 2 (NOX2) is enhanced in cardiomyocytes and macrophages post-MI^83^ and promotes macrophage inflammatory polarization and cytokine expression^84^ and is associated with adverse cardiac remodelling post-MI.^85^ In both human and murine macrophages, miR-106b-5p, miR-148b-3p, and miR-204-5p were shown to suppress NOX2 and targeted up-regulation of these miRNAs through pH-sensitive polyketal NP-specific delivery to macrophages significantly improved infarct size and cardiac function post-MI in mice, highlighting another potential therapeutic axis for management of MI^86^ (*Figure 3A*).

### 4.3 Myocarditis

Myocarditis is an important cause of sudden cardiac death and heart failure, particularly in young, otherwise healthy patients. A breakdown in tolerogenic immune mechanisms is apparent during myocarditis and may particularly underpin autoimmune-mediated myocarditis. For example, miR-98-5p expression is enhanced in B cells during experimental autoimmune myocarditis (EAM) of mice and acts to suppress regulatory IL-10 expression.^87^ Likewise, polarization of tolerogenic dendritic cells, which promote induction of T_REGS,_ may be inhibited in EAM as a result of miR-223-3p down-regulation, de-repressing NLRP3 inflammasome expression.^88^ However, the study of DCs in this work relies entirely on the use of murine bone marrow-derived dendritic cells generated through *in vitro* culture, potentially limiting conclusions regarding translational relevance in humans. None-the-less, T_REGS_ are generally thought to be cardioprotective during myocarditis while effector CD4^+^ T cells are critical players in myocarditis pathogenesis.^89^ Th_17_ effectors play a particularly pathogenic role in later or chronic stages of autoimmune-related myocarditis progression and in the progression to dilated cardiomyopathy (DCM), while the balance of Th_17_ to T_REG_ cells also serves to modulate myocarditis severity, with reduced T_REGS_ leading to more profound Th_17_ responses and more severe disease.^89^ As reported for inflammatory conditions of other solid organs,^39^ miR-155-5p plays a critical role in regulating Th_17_/T_REG_ balance during murine EAM, in which up-regulation of miR-155-5p in cardiac CD4^+^ T cells following induction of EAM promotes Th_17_ responses, while inhibiting generation and responses of T_REGS._^90^ Additional evidence from Coxsackievirus-B3 murine models of viral myocarditis also supports a pathogenic role for up-regulated miR-155-5p in dysfunctional activation of T cells during acute myocarditis, as well as in promoting monocyte/macrophage cardiac infiltration and inflammatory ‘M1’ cardiac macrophage polarization.^91^^,^^92^ Moreover, up-regulated expression of miR-155-5p, as well as the immune-related miRNAs miR-146b-5p and miR-21-5p were observed in human ventricular septal biopsies of acute viral myocarditis patients.^91^ Targeting miR-155-5p expression may therefore hold therapeutic promise for the treatment of myocarditis in certain settings. However, a pathogenic role for miR-155-5p during myocarditis may not be absolute. Natural Killer (NK) cells are innate mediators of viral clearance and are protective in viral myocarditis, with roles in restricting autoimmune inflammation during EAM also reported.^93^ NK cell activation is positively correlated with expression of miR-155-5p, and promotes NK cell expression of IFNγ, partially through repression of the inositol phosphatase SHIP1.^94^ During murine viral myocarditis, increased NK expression of miR-155-5p and enhanced NK cell activation is apparent—promoting viral clearance and reducing cardiac immune cell infiltration and inflammatory cytokine production^95^ (*Figure 3B*).

### 4.4 Cardiac hypertrophy

Leukocyte infiltration into cardiac tissue exacerbates cardiovascular dysfunction in *in vivo* models of pressure overload-induced hypertrophy. miRNA-155-5p was increased in cardiac biopsies of hypertrophic aortic stenosis patients and correlated negatively with cardiac function yet positively with wall thickness relative to non-hypertrophic controls undergoing coronary bypass artery grafting.^41^ Furthermore, in two different murine models of pressure overload, genetic ablation, or pharmacological inhibition of miR-155-5p led to a decrease in cardiac infiltrating leukocytes, particularly CD68^+^CD11b^+^F4/80^+^ monocytes/macrophages. This was accompanied by preserved cardiac function and the absence of hypertrophy, both at the physiological and gene level. These affects were shown to be macrophage specific, as adenoviral-mediated inhibition of miR-155-5p in cardiomyocytes of WT mice did not prevent hypertrophy, whilst in bone marrow adoptive transfer experiments, the presence of miR-155^−/−^ macrophages in WT mice was able to protect against hypertrophy. SOCS1 was the main target of miR-155-5p in macrophages postulated to drive hypertrophy; in the absence of miR-155-5p, SOCS1 overexpression inhibited the JAK/STAT signalling axis and specifically inhibited STAT3 signalling, suppressing the macrophage inflammatory response and release of paracrine mediators driving myocyte hypertrophy.^41^ See *Figure 3C*.

### 4.5 Heart failure

An ongoing inflammatory response is associated with chronic HF. HF patients have elevated levels of circulating pro-inflammatory cytokines including IL-6, IL-1β, and TNFα, and recent analysis of the results of the CANTOS phase III clinical trial revealed that use of the anti-IL-1β monoclonal antibody canakinumab (Ilaris), reduced hospitalizations and mortality related to HF in MI-patients.^96^ However, other phase III trials aimed at modulating HF with anti-inflammatory interventions have had less successful outcomes, suggesting that inflammatory responses may only drive HF in certain patient cohorts.^97^ In DCM patients, CD4^+^ T cell over-activation and proliferation is associated with down-regulation of miR-451a-5p and increased expression of its target Myc,^98^ marking the importance of T cell responses and their regulation by miRNAs in the development of HF. Immunosenescence is also related to the development of HF associated with aging. Ageing-associated miRNAs, such as miR-181 family members—which are important positive regulators of B-cell lymphopoiesis,^20^ are reduced in immune cells of aged patients^45^ and miR-34a-5p which inhibits B cell maturation and demonstrates enhanced expression in leucocytes with aging^99^^,^^100^ may underlie these effects.^46^ Targeting these molecules may mitigate aging-associated HF progression by restoring lymphocyte function. See *Figure 3D*.

### 4.6 Vasculitis

Vasculitis covers a highly heterogeneous group of multi-system autoimmune, inflammatory conditions that affect blood vessels. Although rare, complications of vasculitides can include systemic emboli, thrombosis, aneurysms, sepsis, vessel occlusion, and major end-organ damage. In Wegener’s granulomatosis—the most common form of anti-neutrophil cytoplasmic autoantibody-associated vasculitis, accumulation of effector CD4^+^ T cells and a T-helper profile skewed towards IL-17 and IL-23 producing Th_17_ cells^101–103^ alongside a reduction in T_REGS_ suppressive capacity^104^^,^^105^ is thought to critically regulate disease pathogenesis. In this setting, aberrantly up-regulated expression of miR-142-3p may underlie defective T_REGS_ suppressive activity, potentially driving pathogenic effector T cell responses. miRNA expression profiling of a circulating memory-like population of T_REGS_ found to be enriched within the CD4^+^ T cell pool of patients relative to healthy controls revealed miR-142-3p to be significantly up-regulated alongside decreased expression of the miR-142-3p target adenylate cyclase 9 (ADCY9) and a corresponding reduction in intracellular cyclic adenosine monophosphate (cAMP)^106^—a central mediator of T_REG_ suppression of effector T cells, synthesized by ADCY9. Dysregulated T_REGS_ activity alongside aberrant changes in T_REGS_ miRNA expression has also been reported in Kawasaki’s disease (KD)—an acute form of systemic vasculitis, which can affect the coronary arteries. The proportion of circulating T_REGS_ and T_REGS_ expression of markers of maturation and suppressive function were significantly lower in KD patients (aged 3–54 months), while miR-155-5p and miR-21-5p expression was reduced and miR-31-5p expression was increased significantly.^107^ Putatively, these miRNA expression changes during KD lead to reduced proportion and activity of T_REGS_ through de-repression of the miR-155-5p target SOCS1-inhibiting STAT5 signalling critical for T_REGS_ homoeostasis and function, and restricted expression of the T_REGS_ master transcription factor FOXP3.^107^^,^^108^

## 5. Immune cell-derived EVs and miRNAs—implications for CVD pathogenesis

EVs are a heterogeneous group of lipid-bilayer encapsulated particles, naturally shed into the cellular microenvironment from the cell membrane. Once considered merely a disposal route for unwanted cellular components and debris, it is now appreciated that although this may be the case for a subset of specialized EVs (apoptotic bodies), EVs (including exosomes and microvesicles) can also be packed with numerous forms of viable cargo derived from their parental cells, including intracellular proteins, DNA, and multiple RNA species, such as miRNAs.^109^ Following uptake by recipient cells, molecular cargo delivered within these packages can exert important biological functions, including post-transcriptional gene regulation by non-host cell-derived miRNAs, forming a critical component of intercellular communication and regulatory networks.

**Figure 4:**
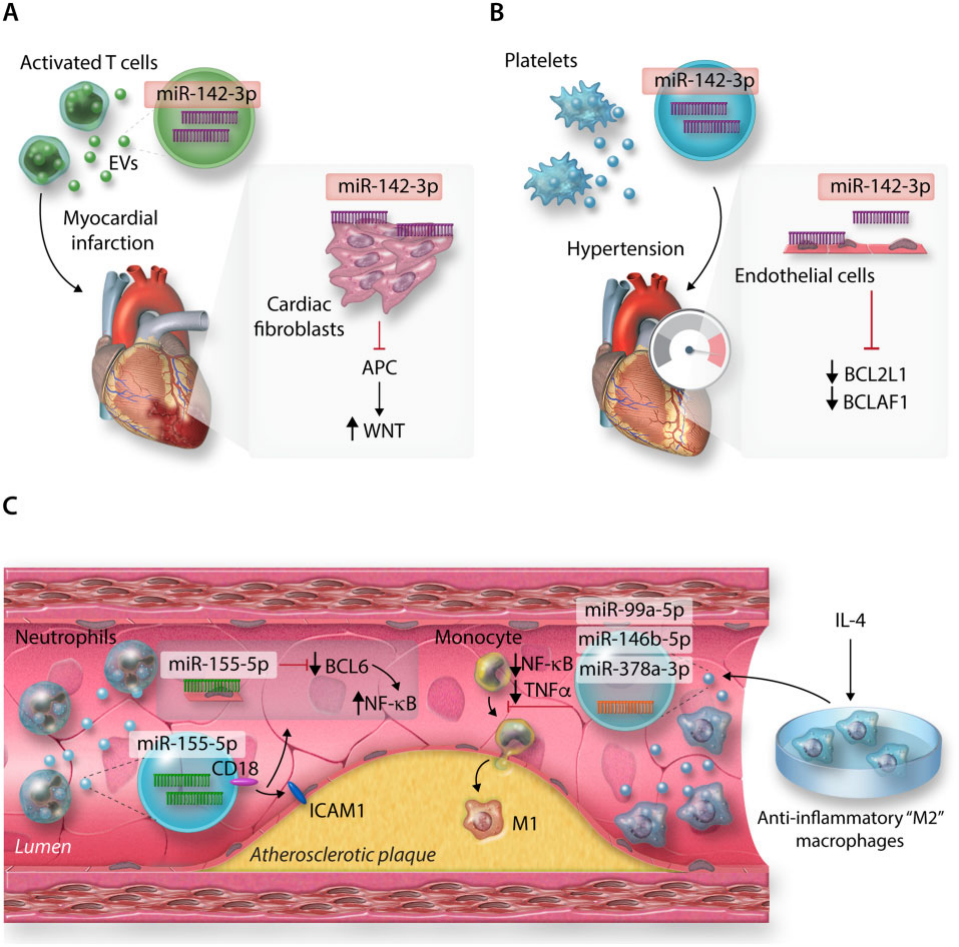
MicroRNAs from immune cell-derived extracellular vesicles (EVs) and their targets in (*A*) cardiac fibroblasts post-MI, (*B*) Endothelial cells (ECs) in hypertension, (*C*) Atherosclerotic plaques.

In the context of CVD, cardiovascular non-immune cells can release EVs loaded with miRNAs to influence intercellular communication and regulation of other cell types, affecting multiple facets of their responses including cell migration and tissue influx, and inflammation.^110^^,^^111^ However, immune cells also utilize these pathways to modulate the surrounding cardiovascular tissue environment^112^ (*Figure 4*). For instance, EVs derived from activated CD4^+^ T cells may be packaged with miR-142-3p.^113–116^ When delivered into mouse hearts following experimental MI, T-cell-derived EVs worsened post-MI cardiac dysfunction via the repression of the miR-142-3p target, adenomatous polyposis coli in cardiac fibroblasts, stimulating up-regulation of WNT-signalling, and pro-fibrotic pathways^116^ (*Figure 4A*). Platelet-derived EVs, thought to be responsible for the majority of circulating EVs,^117^ may also play an important role in CVD pathogenesis via miR-142-3p delivery to cardiovascular cell types. Previously, it has been demonstrated that miR-142-3p plays critical roles in generation of platelets, via the regulation of a network of actin cytoskeletal targets with critical roles to play during megakaryopoiesis.^118^ However, as for T cells, platelet-derived miR-142-3p can extend its regulatory actions beyond merely cell-endogenous effects; in the context of hypertension, ECs may take-up platelet-derived EVs, resulting in abnormal EC proliferation^119^ and apoptosis^120^ via regulation of BCL2-like1^119^ and BCL2-associated transcription factor 1 by platelet-derived miR-142-3p^120^ (*Figure 4B*).

As EVs are secreted into the extracellular environment and may be carried in the circulation, this also raises the possibility that immune cell types not overtly present in cardiovascular tissues in the context of inflammation, may significantly contribute to CVD progress. For example, neutrophils are not typically enriched within atherosclerotic plaques, but may still influence disease pathogenesis through the provision of integrin beta-2 (CD18)^+^ EVs containing neutrophil-derived miR-155-5p (among other miRs), which targets BCL-6 to promote pro-inflammatory NF-kB signalling in ECs at atheroprone arterial regions in a murine model of atherosclerosis^121^ (*Figure 4C*). Additionally, the therapeutic potential of utilizing immune cell-derived EVs to deliberately target pathways involved in CVD pathogenesis has recently become a point of interest.^112^^,^^122^ For example, EVs derived from macrophages polarized towards anti-inflammatory, ‘M2’ phenotypes by IL-4, enriched with miR-99a-5p, miR-146b-5p, and miR-378a-3p may restrict plaque development and pro-inflammatory polarization of circulating monocytes and macrophages in the *Apoe*^−/−^ model of murine atherosclerosis, via down-regulation of NF-kB and pro-inflammatory TNF-α production^123^ (*Figure 4C*).

However, it is important to note that, despite the large number of studies that support a functional capacity for EV-delivered miRNAs to regulate critical cellular pathways, there persists a significant degree of controversy and debate surrounding these claims.^124–126^ For instance, the majority of EVs may actually hold very few copies of individual miRNAs, and the miRNA containing EVs represent only a very small fraction of the total circulating miRNA in plasma.^125^ As such, work in the EV field has been greatly criticized for not, in general, considering the physiological importance of establishing the stoichiometric requirements between the delivered miRNAs and the predicted targets of relevance, in order for a functional impact upon target expression levels to be seen. It is unlikely that EVs carrying very few copies of a specific miRNA will functionally impact the expression levels of a high abundance target in another cell type, unless these EVs are delivered and taken up by target cells in sufficiently large quantities to do so. Additionally, few, if any standardized methods have been agreed on by the field for conducting these studies.

## 6. T_REGS_ immunotherapy and miRNAs: therapeutic potential of miRNA regulated pathways for the treatment of CVD

There has been significant investment in research to target and/or utilize T_REGS_ for treatment of conditions with underlying immunological dysfunctions. This has included graft-versus-host disease (GvHD),^127^ autoimmune conditions, such as Crohn’s disease and diabetes,^128^^,^^129^ and in the context of solid organ transplantation wherein induction of long-term tolerance to the allograft is a major clinical goal.^129^ As it has become clear that T_REGS_ can serve many cardioprotective and vasoprotective roles during CVD, and accumulated evidence also implicates dysregulated T_REGS_ numbers and/or suppressive functions in CVD pathogenesis,^51^^,^^89^^,^^90^^,^^104–107^^,^^130–132^ immunotherapy delivered by exogenously delivered T_REGS_ or through targeted T_REGS_-specific therapies might be a novel therapeutic option.^133–135^

**Figure 5:**
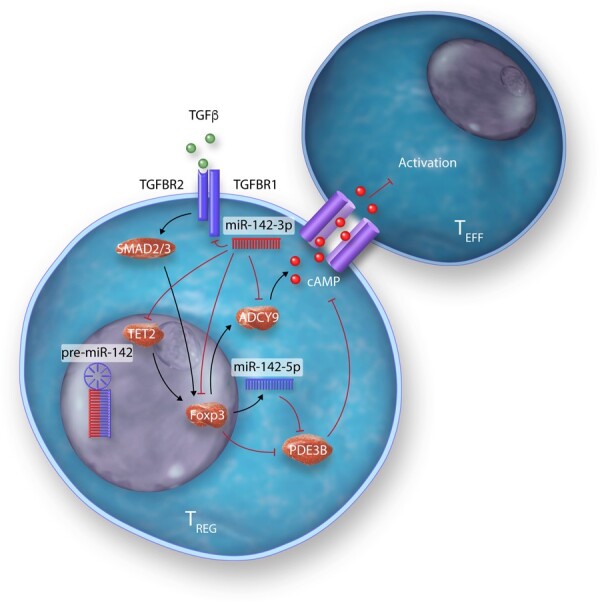
Modulation of multiple biological pathways in T_REGS_ by miR-142 isoforms.

Many studies have identified specific miRNAs, which critically regulate development, differentiation, and the suppressive actions of the spectrum of T_REGS_ subsets identified—from thymus-derived ‘natural’ T_REGS_ (tT_REGS_ or nT_REGS_) to T_REGS_ induced from naïve T cell precursors in the periphery (pT_REGS_) or differentiated *in vitro* (iT_REGS_), in addition to unique T_REGS_ from specialized niches, such as those found in umbilical cord blood T_REGS_ (*Table 2*). Of the miRNAs, which regulate T_REGS_, miR-142 isoforms particularly appear to regulate many facets of T_REGS_ biology (*Table 2* and *Figure 5*). A central mediator of T_REGS_ suppression of effector T cells, cAMP production is critically regulated on several molecular levels by miR-142 isoforms in T_REGS_. Maintenance of ADCY9 expression through the specific down-regulation of miR-142-3p in T_REGS_ constitutes one mechanism of ensuring cAMP production and T_REGS_ suppressive activity.^106^^,^^141^ In addition, our group have also recently demonstrated that a major role for the miR-142-5p isoform in T_REGS_ is to ensure complete suppression of the potent cAMP hydrolase, phosphodiesterase 3B (PDE3B).^26^ In the absence of T_REGS_-specific miR-142-restriction of PDE3B, a severe breakdown in peripheral immune tolerance occurs, resulting in multi-organ tissue damage and widespread immune cell activation in mice.^26^ Conversely, we also recently identified miR-142-3p as a critical regulator of TGF-β mediated T_REGS_ development through the regulation of TGFBR1, and established that deletion of the *Mir142* locus promotes indefinite tolerance to cardiac allografts in a major MHC-mismatched murine model of heterotopic heart transplantation.^27^

**Table 2:** Summary of miRNAs and identified targets involved in regulation of T_REGS_ biology

MicroRNA	Organism studied	T_REGS_ subset investigated	Identified target/s	Altered microRNA expression (↑↓)	Effect of altered microRNA expression on T_REGS_	Ref.
miR-15a-5p/16-5p	*Mm*	CB T_REGS_	FOXP3	**↑**	Forced overexpression suppresses FOXP3 and reduces cellular suppressive functions.	^136^
miR-17-5p	*Mm*	iT_REGS,_ pT_REGS_	EOS, IRF4, SATB1	**↑**	IL-6 promotes miR-17-5p, which suppresses identified targets, compromising FOXP3 function and leading to pro-inflammatory cytokine expression and loss of suppressive activity.	^137^
miR-24-3p	*Hs*	pT_REGS_	FOXP3	**↓**	miR-24-3p is naturally lowly expressed in T_REGS_. Enforced expression reduces FOXP3 expression.	^138^
miR-31-5p	*Hs, Mm*	CB/nT_REGS,_ pT_REGS_	FOXP3	**↑**	miR-31-5p expression is low in T_REGS_—enforced expression reduces FOXP3 expression and TREGS differentiation.	^108^ ^,^ ^139^
miR-125a-5p	*Mm*	pT_REGS,_ iT_REGS_	STAT3, IFNG, IL13	**↓**	Loss of miR-125a-5p reduces T_REGS_ and promotes inflammatory responses, worsening colitis, and EAE. miR-125a stabilizes T_REGS_ homoeostasis.	^140^
miR-142-3p	*Mm* *Hs, Mm* *Hs, Mm* *Hs, Mm*	pT_REGS,_ iT_REGS_ pT_REGS_, iT_REGS_ pT_REGS_ iT_REGS_	TGFBR1 TET2 ADCY9 KDM6A	**↓** **↓** **↑** **↓**	Deletion of *Mir142* in T cells prevented MHC-mismatched heart and skin allograft rejection by augmenting intragraft T_REGS_ via up-regulation of TGFBR1. Decreasing miR-142-3p up-regulated during pancreatic islet autoimmunity, restores T_REGS_ induction and stability during disease. Raised miR-142-3p inhibits T_REGS_ suppressive activity via reduced ADCY9 and cAMP synthesis. miR-142-3p knockdown led to increased FOXP3 expression and suppressive function via up-regulation of KDM6A and promotion of BCL-2 via demethylation of H3K27me3.	^27^ ^28^ ^106^ ^,^ ^141^ ^142^
miR-142-5p	*Mm*	pT_REGS_	PDE3B	**↓**	T_REGS_ specific loss of *Mir142* resulted in loss of peripheral tolerance, widespread systemic autoimmunity, and inflammation due to reduction in cAMP and loss of suppressive function.	^26^
miR-146a-5p	*Mm*	pT_REGS_	STAT1	**↓**	Loss of miR-146a-5p in T_REGS_ leads to breakdown in tolerogenic mechanisms and promotes Th_1_.	^143^ ^,^ ^144^
miR-146b-5p	*Hs*	tT_REGS_	TRAF6	**↓**	Antagomir-knockdown of miR-146b-5p promotes tT_REGS_ function and inhibitory function in GvHD.	^145^
miR-155-5p	*Mm*	pT_REGS_	SOCS1	**↓**	FOXP3 promotes T_REGS_ miR-155-5p expression. Genetic loss of miR-155 suppressed STAT5 signalling and reduced T_REGS_ numbers and homoeostasis.	^40^
miR-181a/b-5p	*Mm* *Mm*	tT_REGS_ iT_REGS_	CTLA4 SMAD7	**↓** **↑**	Loss of miR-181a/b-5p restricts thymic T_REGS_ development but promotes peripheral TREGS suppressive activity. Transfected miR-181a-5p mimics enhance FOXP3 expression and T_REGS_ differentiation.	^146^ ^147^
miR-202-5p	*Hs*	pT_REGS_, iT_REGS_	MATN2	**↑**	During allergic rhinitis, up-regulated miR-202-5p inhibits T_REGS_ development and function.	^148^
miR-210-3p	*Hs*	pT_REGS_	FOXP3	**↓**	miR-210-3p is naturally lowly expressed in T_REGS_. Enforced expression reduces FOXP3 expression.	^138^
miR-340-5p	*Mm*	pT_REGS_	IL4	**↓**	CD226 deficiency in TREGS promoted Th2, by reducing miR-340-5p, promoting T_REGS_ IL-4 release.	^149^
miR-1224-5p	*Mm*	pT_REGS_	FOXP3	**↓**	AhR signalling attenuates pertussis toxin-induced systemic inflammation by suppressing miR-1224-5p, enhancing FOXP3 expression, and promoting T_REGS._	^150^
miR-4281-3p	*Hs*	iT_REGS_	FOXP3	**↑**	Intranuclear localization and binding of miR-4281-3p to FOXP3 promotor TATA-box enhanced FOXP3 expression, T_REGS_ differentiation, stability, and function.	^8^

CB, cord blood; *Hs, Homo sapiens*; i/n/p/t T_REGS_, inducible/natural/peripheral/thymic regulatory T cells; *Mm, Mus musculus*.

However, what these findings and much of the collective information regarding miRNA regulation of T_REGS_ mean in the context of CVD largely remains to be investigated. Indeed, despite good evidence for protective functions of T_REGS_ during CVD, studies that characterize the potential for T_REGS_ therapy in major forms of CVD, such as atherosclerosis, are currently lacking–as recently outlined by Albany *et al*.^135^ The concept of using miRNA mimics and/or synthetic miRNA inhibitory molecules (i.e. antagomiRs) as therapeutic agents is highly topical.^151^^,^^152^ Candidates in early development aim to target a range of conditions including hepatitis C infection (Mirvirsen, RG-101), cancer (MesomiR, MRG-106), and ischaemia (MRG-110), with promising results in phase I and II clinical trials. Furthermore, pre-clinical data using antagomiR-treated T_REGS_ in humanized mouse models of GvHD indicate that *ex vivo* miRNA modulation of T_REGS_ is a promising avenue to enhance T_REGS_ stability and suppressive activity during adoptive cell transfer immunotherapy.^145^ The prospect that targeted miRNA manipulation of T_REGS_ might overcome some of the predicted limitations of adoptive transfer T_REGS_ immunotherapy,^129^ such as stabilization of specific regulatory phenotypes or better control over trafficking and migration to desired sites, is particularly enticing both for CVD and other conditions. In addition, these points also raise the possibility that correction of dysfunctional T_REGS_ in specific forms of CVD might also be achieved therapeutically by targeting aberrantly expressed miRNAs in *ex vivo* expanded populations of the patient’s cells. This may be particularly important when considering that, although interventions targeted to cardiovascular resident or infiltrating cell populations may be desirable for treatment of CVD, off-target, systemic suppression of immunity and inflammatory responses may theoretically result in unwanted general and/or opportunistic infections and other undesirable outcomes—despite any benefits to CVD treatment.

## 7. Concluding remarks

Attempts to characterize and better understand how immune system responses are regulated by miRNAs is a highly topical avenue of research. Unravelling the dynamics of these regulatory pathways and their functions in specific immune and inflammatory cell types is key to their therapeutic potential. With regards to CVD, it is critical to examine the dynamics of immune system regulation by miRNAs in each specific disease context, rather than make generalized inferences. Against this background, for certain well-known immuno-miRNAs, such as miR-155-5p, there is a robust body of literature, which implicates dysregulated expression in CVD pathogenesis. In our opinion, this molecule represents an obvious therapeutic candidate target of interest in several forms of CVD. However, for other immuno-miRNAs, there are notable gaps in the literature surrounding immune system regulation specifically in CVD. For example, the mature isoforms of miR-142 demonstrably influence key immune system developmental and cellular signalling pathways, and circulating levels are altered in CVD,^153–155^ but they have rarely been directly investigated in this setting.

As discussed in this review, among leucocytes, macrophages and CD4^+^ T cell responses are particularly important in CVD aetiology and progression. Targeting miRNAs and miRNA-regulated signalling networks in these cells—particularly those which regulate multiple aspects of immune cell biology, may prove to be especially useful in immunotherapeutic settings. However, how best to target these molecules in an effective, safe, yet cell type-specific manner is still unclear. The use of modified synthetic anti-sense oligonucleotides, such as antagomirs may be associated with off-target effects, particularly at higher therapeutic doses *in vivo*. Additionally, such effects could be relatively long-lasting, given the enhanced stability of these molecules. Packaging technologies, such as modified polymeric NPs may overcome some of the challenges the field faces, for instance, allowing for more controlled delivery or release of therapeutic molecules to target cells.^156^ As we have outlined, an alternative to this cell-targeted approach may be to turn to immunotherapy using miRNA modified T_REGs_ to suppress dysregulated inflammation and immune responses underlying CVD pathogenesis. This area warrants further investigation and significant research effort.

Finally, recent advances in single-cell sequencing and analytical technologies allow for a depth in the resolution of data concerning rare cardiac immune cell populations, previously not accessible to researchers. For instance, it has only recently come to light that a population of tissue-resident, helper-like innate immune cells (ILCs) displaying a predominantly group 2-commited profile (ILC2) exists within heart tissue in mice and humans. These cells may play important roles during CVD including myocarditis, cardiac ischaemia, and pericarditis,^157–159^ while populations observed in para-aortic AT and lymph nodes may also serve vital roles in directing progression of atherosclerosis.^160^ However, these cells have primarily been studied in mice thus far and the relevance to human CVD remains to be established. Although still in its infancy, our understanding of how miRNAs regulate development, homeostasis, and activity of ILCs is gradually being unravelled.^161–163^ Future studies should aim to identify how these molecules regulate these newly identified populations, both during health and disease. This may potentially reveal exciting new avenues of therapeutic promise for patients suffering from CVD.

**Conflict of interest**: A.M.S. is a consulting editor for Cardiovascular Research. G.M.L. has filed a patent regarding modulation of PDE3B by miR-142.

## Funding

L.B.R. is supported by the National Institute for Health Research (NIHR) Biomedical Research Centre based at Guy's and St Thomas' NHS Foundation Trust and King's College London. P.K. is supported by a King’s British Heart Foundation (BHF) Centre PhD Studentship. J.K.H. is supported by the Medical Research Council (grant MR/K002996/1). A.M.S. is supported by the BHF (CH/1999001/11735, RE/18/2/34213), the NIHR at Guy’s & St Thomas’ NHS Foundation Trust in partnership with King’s College London and King’s College Hospital NHS Foundation Trust (IS-BRC-1215-20006), and the Fondation Leducq. G.M.L. is supported by the Wellcome Trust (grant number 091009), the British Heart Foundation (award number PG/12/36/29444), the MRC (grant number MR/M003493/1), and the NIHR at Guy’s & St Thomas’ NHS Foundation Trust in partnership with King’s College London and King’s College Hospital NHS Foundation Trust.
